# Arsenic in Surface Soils Affected by Mining and Metallurgical Processing in K. Mitrovica Region, Kosovo

**DOI:** 10.3390/ijerph7114050

**Published:** 2010-11-18

**Authors:** Trajce Stafilov, Milihate Aliu, Robert Sajn

**Affiliations:** 1 Institute of Chemistry, Faculty of Science, Sts. Cyril and Methodius University, POB 162, 1001 Skopje, Macedonia; 2 Faculty of Medicine, Prishtina University, Bulevardi i deshmoreve, Prishtina, 10000, Kosovo; E-Mail: melihatealiu@hotmail.com; 3 Geological Survey of Slovenia, Dimiceva ul. 14, 1000 Ljubljana, Slovenia; E-Mail: Robert.Sajn@geo-zs.si

**Keywords:** arsenic, soil, pollution, K. Mitrovica, Republic of Kosovo

## Abstract

The results of a study on the spatial distribution of arsenic in topsoil (0–5 cm) over the K. Mitrovica region, Kosovo, are reported. The investigated region (300 km^2^) was covered by a sampling grid of 1.4 km × 1.4 km. In total, 159 soil samples were collected from 149 locations. Inductively coupled plasma–mass spectrometry (ICP-MS) was applied for the determination of arsenic levels. It was found that the average content of arsenic in the topsoil for the entire study area was 30 mg/kg (from 2.1 to 3,900 mg/kg) which exceeds the estimated European arsenic average in topsoil by a factor of 4.3. Contents of arsenic in the topsoil exceeded the optimum value recommended by the new Dutchlist (29 mg/kg As) in 124 km^2^. The action value (55 mg/kg As) was exceeded in 64 km^2^, with the average content of 105 mg/kg (from 55 to 3,900 mg/kg As).

## 1. Introduction

There are many different sources of heavy metal contaminants, including mining and metallurgical industries [1]. Heavy metals, which are known to have adverse effects on the environment and human health, are an especially dangerous type of contaminant because of their persistence and toxicity. They are significantly toxic even in small amounts and can cause diseases in humans and animals as they cause irreversible changes in the body, especially in the Central Nervous System [2].

Arsenic in Nature usually occurs in the form of sulphides such as arsenopyrite, FeAsS, realgar, As_4_S_4_, orpiment, As_2_S_3_, and arsenopyrite, FeAsS [3]. Large exposure to As by ingestion can cause problems in the digestive and nervous systems and to the activity of the heart [2,4]. Contamination of the environment by arsenic from both anthropogenic and natural sources has occurred in many parts of the World and is now recognized as a global problem [5,6]. The principal anthropogenic sources for soil contamination by As include base metal smelters [7–15] and the mining of arsenic [8,16–18], lead and zinc [7,10,11,15,19–21], gold [18,22,23] and other types of mines [24–30]. Power plants that burn As-rich coals or treated lumber, disposal sites for wastes from As-processing plants, and industrial and municipal dump sites also represent sources of arsenic contamination in soil [1,5].

Mining and metallurgic activities in Kosovo have a long history. Trepča Mine Limited in Mitrovica was built in 1927 and produced lead, zinc, arsenic and cadmium from the 1930s until 2000. The smelter close to Zvečan commenced working in 1939. Because of the smelter and the three tailing dams of the factory, environmental pollution in Mitrovica increased dramatically. The smelter has worked sporadically since the 1999 conflict in Kosovo. However, an environmental audit ordered by UNMIK and conducted in March and April 2000 warned that it should be closed down as it was an unacceptable source of air pollution [31–33]. The total production at Trepča from 1931 to 1998 is estimated as being 34,350,000 t run-of-mine ore at grades of 6% Pb, 4% Zn, and 75 g/t Ag and 102 g/t Bi. The ore was used in the Prvi Tunel flotation with a capacity of 760,000 t/y. The lead concentrates were brought to the lead smelter of Zvečan (capacity 80,000 t/y) and the zinc concentrates to the zinc smelter of K. Mitrovica (capacity 50,000 t/y). There was also a unit for the production of fertilizers using the sulphuric acid by-product of hydrometallurgy, and lines of battery production and battery recycling. The metal production was 2,066,000 t Pb, 1,371,000 t Zn, 2,569 t Ag and 4,115 t Bi. Gold production was estimated at 8.7 t from 1950 to 1985, *i.e.*, an average of 250 kg/y, and Cd production was estimated at 1,655 t from 1968 to 1987. Traces of Ge, Ga, In, Se and Te in the run-of-mine ore have also been reported, which were valorized at the smelter level [31–33].

Ore processing in the Zvečan smelter released large quantities of particulate and gaseous emissions from two stacks and produced large volumes of mining and metallurgical wastes that accumulated in large dumps. At the end of the 1990s, the overall emission of dust from the Zvečan smelter was estimated at 730 t/year, and over time about 40,000,000 tons of tailings accumulated in the Zvečan area [34]. Air-borne stack emissions, mine dump drainage and wind-borne mining wastes were the major sources of a widespread diffusion of heavy elements into the environment. This affected the distribution and behaviour of various chemical species in the surface geochemical spheres and resulted in very heavy pollution of the area around the Ibar and Sitnica valleys [34].

The effect of mines and mining industries on the environment in Kosovo is difficult to ascertain as little data exist since 1999 due to the war in Kosovo. Problems range from hazardous materials to air/soil/water pollution. Several reports have indicated that the current levels of lead exposure are extremely high in the soil and in the air as well [35–42].

**Figure 1:**
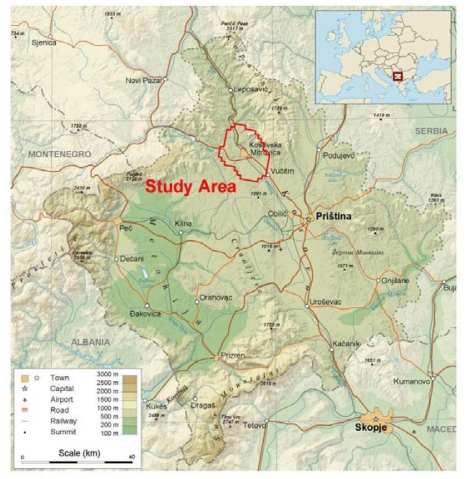
Location of the study area.

**Figure 2:**
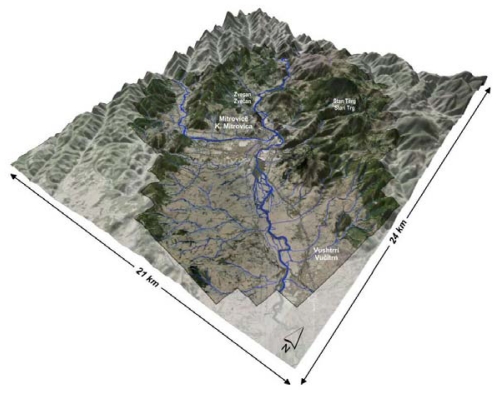
Digital elevation model of the study area.

The main objective of the present investigation was (i) to determine the content of arsenic, an essential elements, but toxic in high concentrations, (ii) which minerals are present in the lead and zinc ores from these region [37,43–47], (iii) to establish the spatial distribution of arsenic in soils from the broad area of K. Mitrovica (Figures 1 and 2) and (iv) to assess the size of the area affected by the mining activities and by the smelter plant situated nearby.

## 2. Materials and Methods

**Figure 3:**
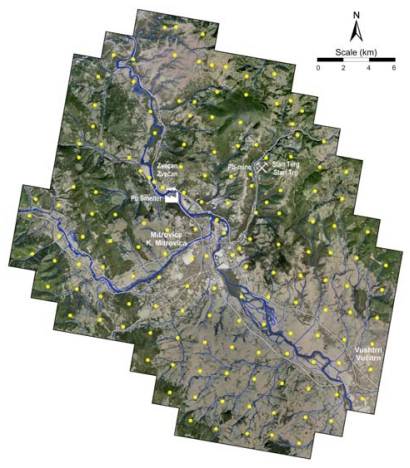
Sampling locations (given in red points).

Sampling was carried out from January to May 2009. Soil surface samples (0 cm to 5 cm depth) were collected in the town of K. Mitrovica and its surrounding region (Figures 2 and 3). In total, 159 samples were collected from 149 locations, including locations near the mining centres of K. Mitrovica. The whole of the region investigated (300 km^2^) was covered with a sampling grid of 1.4 × 1.4 km^2^ (Figure 3). Samples were located using the Global Positioning System (GPS), topographic maps at scale of 1:25,000 and Google Earth maps (http://earth.google.com/). Each sample represented the composite material collected at the central sampling point itself together with at least four points collected around a central one with a radius of 50 m towards N, E, S and W directions. The composite material of each sample (about 1 kg) was placed into plastic self-closing bags and brought to the Institute of Chemistry, Faculty of Science, University of Skopje, Republic of Macedonia, where they were prepared for atomic spectroscopy.

All of the collected soil samples were then shipped to the ACM Analytical Laboratory in Vancouver, BC, Canada. Analyses were conducted using mass spectrometry with inductively coupled plasma (ICP-MS) after Aqua Regia Digestion (the 1DX1 and DISP2 methods).

All samples (n = 156), replicates (n = 6) and geological standards (n = 8) were submitted to the laboratory in a random order. This procedure assured an unbiased treatment of samples and a random distribution of the possible drift of analytical conditions for all samples. Eleven randomly selected samples were replicated for precision estimation. The geological standard materials DS7 (n = 4) and OREAS45PA (n = 4) were used for estimating precision. The sensitivity in the sense of the lower limit of detection for As was found to be 0.1 mg/kg. The precision was also tested by the Thompson-Howarth method and it was found to be less than 5%.

## 3. Results and Discussion

Arsenic is in the group of elements toxic to humans [48]. Once arsenic is in the body, it binds to haemoglobin, plasma proteins, and leukocytes and is redistributed to the liver, kidney, lungs, spleen, and intestines. Arsenic causes cellular damage through a variety of mechanisms. Arsenic binds to enzyme sulfhydryl groups and forms a stable ring that deactivates the enzyme. The process of deactivating the enzyme causes widespread endothelial cell damage, vasodilatation, and leakage of plasma. Massive transudation of fluid into the bowel lumen, mucosal vesicle formation, and tissue sloughing may result in large gastrointestinal fluid losses. Arsenic binds to dihydrolipoic acid, a pyruvate dehydrogenase cofactor, blocking the conversion of pyruvate into acetyl coenzyme A and inhibiting gluconeogenesis. Long-term exposure results in nerve damage and may lead to lung, skin, or liver cancer.

**Figure 4:**
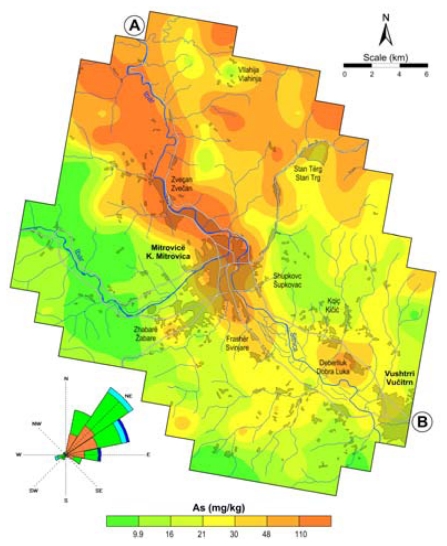
Spatial distribution of arsenic in topsoil.

**Figure 5:**
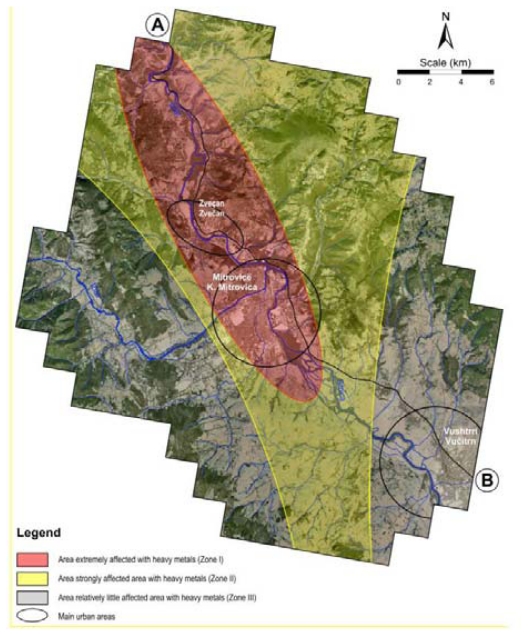
Location of the determined polluted zones, main urban areas and cross-section (profile A–B).

**Figure 6:**
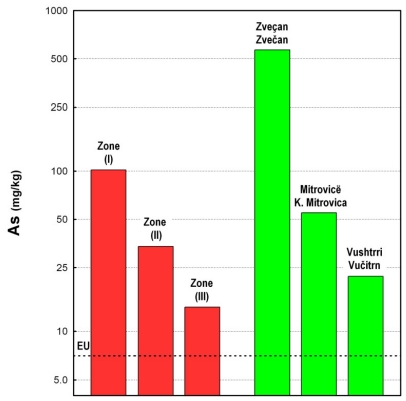
Distribution of arsenic in topsoil according to determinate polluted zones and main urban areas.

The average amount of As in soils throughout the World is 5 mg/kg [49], and in European topsoil it is 7.0 mg/kg [50]. The geometric mean of As in the topsoil for the entire study area was 30 mg/kg (median value of 25 mg/kg), with a range of 2.1–3,900 mg/kg (Table 1). This means that the arsenic average for the whole area exceeds the estimated European As average in topsoil by a factor of 4.3. It is evident from the obtained results (Table 2, Figure 4) that the content of arsenic is very high in the topsoil in the areas of the lead and zinc smelter plants (cities of Zvečan and K. Mitrovica) (Figures 5 and 6).

As can be seen from Figure 4, the content of arsenic in topsoil in the areas near the lead and zinc smelter plant (*i.e.*, the cities of Zvečan and K. Mitrovica) is high, but it is also high in the topsoil on the northern site of the smelter plant, as well as in the area of mining activities on the north-eastern part, following the wind rose (Figure 4). Therefore, the study area was divided into three zones (Table 2, Figures 5 and 6): Zone I (the most polluted area), Zone II (medium polluted area) and Zone III (non-polluted area). As can be seen, the most polluted area (Zone I) had an average As content of 100 mg/kg, in Zone II the average value was almost three times less (34 mg/kg) and in the zone III the As content in the soil was much lower (14 mg/kg). In the area of the city of Zvečan (Table 2, Figures 5 and 6), the average content of As in five samples reached the extremely high value of 570 mg/kg, whereas in the samples from the city of K. Mitrovica the average value was much lower (55 mg/kg) and it was evident that the soil samples from the city of Vučitrn were not strongly polluted with As (22 mg/kg), although they still exceeded the European topsoil average [50] by more than three-fold. According to these data, it is evident that the source of the high arsenic contents in this region is the lead and zinc smelter plant.

**Figure 7:**
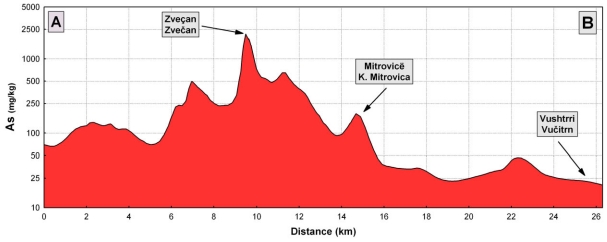
Distribution of arsenic in topsoil (cross-section, profile A–B).

**Figure 8:**
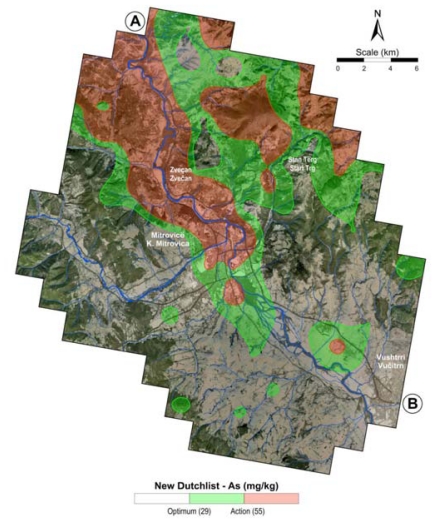
Spatial area pollution with As in topsoil according to the New Dutchlist recommendation.

In the regions of Zvečan and K. Mitrovica, several topsoil samples with very high contents of arsenic were found. It should be noted that sample No. 91 (N of Zvečan) had a content of 3,900 mg/kg, which is 557 times higher than the European topsoil average [50]. This value is clearly presented in Figure 7, as well as in Figures 4 and 8, where the distribution of arsenic in topsoil is shown as a cross-section (profile A–B in Figure 5). It can be seen that the high contents of arsenic appear from about 8–10 km north of the smelter plant (Zvečan), and 6 km south of it (just SE of the city of K. Mitrovica).

The contents of arsenic in the topsoil exceed the optimum value recommended by the new Dutchlist (29 mg/kg As) over an area of 124 km^2^ (Figure 8). The main polluted area was established by marking the sites with As contents exceeding the intervention value of 55 mg/kg according to the New Dutchlist (http://www.contaminatedland.co.uk/std-guid/dutch-l.htm). It was found that this main polluted area covers 64 km^2^ (Figure 8) and its average As content is 105 mg/kg (from 55 to 3,900 mg/kg) which is 15 times higher than the European As average (Table 3).

It is evident from these results that arsenic is present in the ore and concentrates treated in the processes in the flotation and smelter plants. The mine at Trepča is a well-known polymetallic mine with more than 60 different minerals [43–45]. In this case, as probably accompanies other sulphide minerals in the form of arsenopyrite. We assumed that most of As is mobilized by air transport through combustion products after ore smelting, but the soil is also partially natural enriched by naturally occurring As. From the results obtained it can be also concluded that the effect of soil pollution and the existence of a permanent source of pollution (flotation tailings and slag deposit in Zvecan) influences the health of the local population and remediation is needed. This is especially important when taking into account the level of pollution when combined with other heavy metals in this area (Pb, Zn, Cd) [51–53].

## 4. Conclusions

The results of this study on the spatial distribution of arsenic in topsoil (0–5 cm) over the K. Mitrovica region, Kosovo, show that the average content of As in the topsoil for the entire study area is 30 mg/kg (with a range of 2.1–3,900 mg/kg), which exceeds the estimated European arsenic average by a factor of 4.3. It is evident that the content of arsenic is very high in topsoils from the areas closest to the lead and zinc smelter plant (Zvečan zone), as well as in topsoils in the city of K. Mitrovica. In these regions, several topsoil samples with extremely high contents of arsenic were found. Contents of arsenic in the topsoil exceed the optimum value recommended by the new Dutchlist (29 mg/kg As) in about 41% of the study area. The action value (55 mg/kg As) was exceeded in about 21% of the study area, with an average content of 105 mg/kg and a range of 55 to 3,900 mg/kg As, which is 15 times higher than the European As average.

## Figures and Tables

**Table 1:** Descriptive statistics of measurements for arsenic in soil (values given in mg/kg).

*N*	*Dis.*	*X_g_*	*s_g_*	*Md*	P10	P90	Min	Max	A	E
156	Log	30	3.1	25	9.2	150	2.1	3900	1.27	2.74

Dis. = distribution (Log = lognormal); X_g_ = geometric mean; s_g_ = geometric standard deviation; Md = median; P_10_ = 10 percentile; P_90_ = 90 percentile; Min = minimum; Max = maximum; A = skewness; E = kurtosis.

**Table 2:** The average content of arsenic in soil in the study area, and then according to the three recognized zones of pollution recognized and the cities of Zvečan, K. Mitrovica and Vučitrn (values given in mg/kg). The EU average is also shown for comparison.

	EU	Study area	Zone I	Zone II	Zone III	Zvečan	K. Mitrovica	Vučitrn
N*	840	156	30	65	61	5	11	8
**As**	7.0	30	100	34	14	570	55	22

* N = number of samples; EU = European average value.

**Table 3:** Statistical data for the main polluted area.

Dutch standard	Permitted, mg/kg	Area, km^2^	Percentage of the total area, %	Average, in mg/kg	Min, in mg/kg	Max, in mg/kg

Optimal value	29	124	41.3	56	29	3,900
Action value	55	64	21.3	105	55	3,900
